# In Service of the Society? Medical Associations as Agents of Social Change—Implications for Health Policy and Education in Israel

**DOI:** 10.3390/healthcare9101264

**Published:** 2021-09-25

**Authors:** Baruch Levi, Nadav Davidovitch, Keren Dopelt

**Affiliations:** 1 Department of Health Policy and Management, School of Public Health, Faculty of Health Sciences, Ben-Gurion University of the Negev, P.O. Box 653, Beer-Sheva 8410501, Israel; Baruchle@post.bgu.ac.il (B.L.); nadavd@bgu.ac.il (N.D.); 2 Department of Public Health, Ashkelon Academic College, Ben Tzvi St. 12, Ashkelon 78211, Israel

**Keywords:** health policy, public health, medical associations, medicalization

## Abstract

This study aims to explore what medical associations in Israel do to promote public health, what values underpin their activities, and how their actions can be interpreted. For this purpose, an analysis of both individual and organizational levels was applied in an effort to yield a more nuanced understanding of the relationship between society and the medical profession. In-depth interviews with senior physicians were conducted, combined with a review of policy and public initiatives of medical associations between 2008 and 2018. The findings of this study reveal that medical associations engage in a range of social and policy initiatives designed to promote public health, but, at the same time, they tend to construct socially related health issues as medical problems in a manner that fits their sectorial agendas. This may reflect organized medicine’s efforts to extend its dominance over society through the application of the biomedical model to social issues. It is necessary to integrate biosocial training with medical education to ensure that future physicians are equipped with the skills needed to implement social medicine.

## 1. Introduction

In recent years, there has been a growing academic interest in the third sector. The vacuum created by the state due to the rise of market ideology and reduction in the public sector was filled by non-profit organizations that took on tasks previously performed by government institutions [1,2]. Healthcare is one of the main fields where this process took place. This is especially true in Israel, where neoliberal ideology began to occupy a central place in the field of socio-economic policy in the mid-1980s [3].

The vague nature of the third sector makes it difficult to reach a uniform and agreed-upon definition. In the most basic sense, the third sector is often defined through negation, as a part of the economy that does not belong to the profit-driven market, nor does it pertain to the government’s bureaucratic chain of command. In this sense, the third sector can be perceived as an umbrella term for a variety of organizations that populate the space of civil society, such as registered charities, community groups, faith-based organizations, professional associations, trade unions, self-help groups, social movements, and advocacy groups [4].

The continuing decline in the share of public funding and the acceleration of privatization and commodification processes of health services were accompanied by an increase in the activity of civil society organizations, which, from the beginning, occupied a prominent place in the Israeli healthcare system [5,6,7]. Nevertheless, the involvement of health professionals in health-related social issues has, to date, received little attention. The role played by the medical profession in this context is particularly interesting in light of physicians’ dominant professional and social status in the healthcare system and wider society. However, little is known about the attitudes and actions of medical associations as agents of social change. Therefore, this study aims to explore what medical associations do in the public sphere to promote public health, what values underpin their activities, and how their actions can be interpreted. Answering these questions has the potential to extend our understanding of medical associations as agents of social change, and, more generally, deepen our theoretical insight concerning the relationship between the medical profession and society.

Medical associations, for the purpose of this study, are the representative organizations of physicians. Their main role is to promote the interests of physicians vis-à-vis employers, insurers and regulators, including negotiating contracts and signing collective bargaining agreements. They also deal with professional, disciplinary and ethical matters related to medicine to various degrees in different countries [8]. Medical associations can function as trade unions, chambers, pressure groups and so forth, depending on their historical development and political environment. Certain types of association focus on the clinical and scientific aspects of specific medical specialties, such as cardiology and oncology (often referred to as “colleges”, “societies” or “academies”).

Israel is a particularly interesting case study, as, traditionally, third-sector organizations have always played a major role in various healthcare domains in Israel [9]. With the withdrawal of the government from domains related to public health, due to the increasing privatization and commercialization of the healthcare system, it seems that the involvement of the third sector in the health sector, as well as in other areas, has intensified [5,10]. Medical associations in Israel have always been involved in various aspects of education and ethics, and in the regulation of the healthcare system. Moreover, they see themselves as representatives of the public vis-à-vis the state, and as advocates of the health system [8,11]. Cooperation between the medical associations and the Ministry of Health and, specifically, with the Public Health Division, occasionally occur, especially in the form of consultations and information exchanges. However, medical associations in Israel function mainly as external pressure groups rather than bodies involved in health policy formation [8].

Therefore, examining their activity in the field of public health, while focusing on their motivations, goals and methods of operation, may shed some light on the relationship between medical profession and society from a perspective that does not usually receive much attention. The insights that emerge from the Israeli case may be relevant to other healthcare systems that struggle with public health issues such as obesity, smoking and health disparities, and where the medical profession is generally involved in health policy issues.

Furthermore, the question of the role played by medical associations as agents of social change is particularly interesting because it sharpens the conflicts and contradictions between different approaches concerning the motivations of political entities such as medical associations. On the one hand, their involvement in the public sphere could be attributed to their sense of moral and professional obligations to society. On the other hand, their actions could also be interpreted as an effort to shape social values and practices in a fashion that accommodates their own interests.

As further illustrated, the literature tends to explain the actions of the medical profession in terms of self-interest. While this is a helpful assumption in many cases, relying on it exclusively leaves very little room for a more complex interpretation of the actions of medical associations in the public sphere. Although altruistic actions are sometimes acknowledged, this is usually at the level of the individual physician and, therefore, often marginalized in the process of policy analysis. Since medical associations consist of many individual physicians expecting their representative organization to carry out their will and desires, it seems that a study that involves both the micro- (individual) and meso- (organizational) level, while trying to reconcile the two approaches—self-interest and professional altruism—may yield a more nuanced view of the medical profession and promote a deeper understanding of the relationship between society and the medical profession. By doing this, we expect to elucidate not only the medical profession’s motivations and spheres of influence, but also its boundaries and limitations as an agent of social change.

### 1.1. A Brief Overview of the Israeli Healthcare System

The National Health Insurance Law (1994) sets a healthcare package that is uniform, equal, and universal to all Israel’s citizens. Pursuant to the law, citizens are insured with mandatory insurance in one of the four health funds that act as non-profit organizations [12]. The health funds’ package includes services such as medications, doctors’ visits, hospitalization services, mental health services, dental care and paramedical treatments. Concurrently, the state funds a separate services package supplied by the Ministry of Health, which includes mental health hospitalization services, geriatrics, preventive medicine, and rehabilitation equipment.

Israel’s healthcare system is comprised of a mix of public and private services, provision and finance. About 64% of the national healthcare expenditure comes from public finance and designated taxing, with the rest coming from private expenses, including both direct payment for healthcare services and private health insurances. The national expenditure on healthcare in Israel is 101 billion Israeli new shekels (NIS), and it constitutes about 7.5% of the gross domestic product (GDP)—a rate that is considered low compared with other developed countries [7].

Israel has 45 general hospitals, 11 of which are government-operated, while 8 belong to the Clalit Healthcare Services health fund, 11 are private and the rest are public institutes affiliated with the Hadassah organization, the Mission, and other organizations. The Ministry of Health, through its Public Health Division, is in charge of the public health. The main activities of this division include routine vaccinations, disease control, family health centers, school health services, health promotion and education. In addition, the Public Health Services Division is currently at the forefront of the fight against the Coronavirus disease 2019 (COVID-19) pandemic in Israel. Health experts believe that public health services in Israel have not been properly budgeted for many years. In contrast with the public health insurance services provided by the health funds, the public health services’ budget is not regularly updated, for example, according to population growth. Therefore, services such as school health services and family health centers generally suffer from workforce shortages and operate under conditions of budgetary uncertainty.

### 1.2. Physicians and Medical Associations as Agents of Social Change

Individuals, groups, and organizations often operate in the space of civil society as agents of social change. They pursue collective goals and engage in various activities of public importance through different means, such as framing the public agenda, intensifying citizens’ awareness of political and societal issues, and trying to influence political decision-making. They do so to help communities overcome certain challenges and improve the lives of their members [1].

In a world where the main causes of morbidity are environmental and social determinants deriving from conditions of life, and access to health services often depends on economic and political agendas [13], one may contend that the involvement of health professionals in public affairs is of the utmost importance.

Indeed, physicians and medical associations worldwide are often involved in broader societal and ethical issues that concern public and individual health. Physicians are often involved in social programs concerning health promotion [14]. Some join Non-Governmental Organizations (NGOs) that advocate human rights [15]. The notion of physicians as health advocates corresponds with the idea that social responsibility is integral to the physician’s role. According to this perspective, in addition to patients who individually seek the physician’s care and attention, the neighborhood, the community, and the nation are all, in essence, the physician’s patients [16]. Not only do physicians tend to be involved in social activities themselves, but they also generally expect and support this type of action from their representative organizations [17,18].

Public roles assumed by physicians could be perceived as an expression of the “social contract” between the medical profession and society, highlighting the moral responsibility of medical practitioners toward society in the broader sense of professionalism, which exceeds the narrow focus on clinical work or purely medical issues. Such activities are perceived by physicians as an opportunity to restore human contact between them and their patients [19,20]. Indeed, the motifs of ethics are deeply embedded in the heart of the medical ethos, as expressed not only in international laws and conventions but also in the education and training of health professionals, as well as the doctor’s oath. This has the potential to spur a leadership that extends beyond the limits of medical institutions. Most notably, Talcot Parsons’ functionalist perspective [21] focused on what may be perceived as the medical profession’s pivotal position in the social system. According to Parsons [21], the importance of professions, with a special emphasis on the medical profession, stems not so much from their intrinsic features but from their distinctive institutional framework. This approach is akin to Durkheim’s conception of professions as a positive force, possessing the potential to unify and contribute to solidarity and the social order in an increasingly complex and fragmented modern society, and thus offering a benevolent portrayal of the medical profession [22,23,24].

From this perspective, it is to be expected that medical associations will also take part in public activities for the common good, and play a role in the creation of healthier, safer and more prosperous societies through activities such as advocacy, realization of rights, provision of services and so forth. Ahlquist and Levi [25] demonstrate how activist labor unions engage in voluntary activities that promote social solidarity beyond the private interests of individual members. Indeed, medical associations across the globe help frame the public agenda, intensify citizens’ awareness of health issues, and try to influence political decision making on diverse matters, ranging from health disparities and social determinants of health to prisoners’ right to medical treatment, torture, and female genital mutilation [26,27,28]. However, from a theoretical perspective, the question of why medical associations would choose to take on such tasks is intriguing, and far from straightforward. One must take caution when simply viewing this activity as a direct manifestation of their members’ wishes.

It seems that the literature on medical sociology tends to distinguish between the aims of and motivations for action at the individual level and the organizational level. Coburn [29], for example, points out that while the voluntarism and social involvement of individual medical professionals receives some recognition in the literature, the actions of the profession as an organized entity are often perceived with some cynicism, as it tends to pursue more mundane interests. Blackmer [30] makes a similar argument, stating that the literature on medical professionalism tends to concentrate on the obligations of the individual physician towards the individual patient, while nearly no consideration has been given to organized medicine in terms of altruism and voluntarism. In other words, the actions of organized medicine are generally viewed through a more utilitarian perspective than one concerning the individual physician.

Scholars often argue that professions in general, and the medical profession specifically, have rightly “earned” their reputation as entities guided by self-interest, pursing economic rewards and status for themselves. Seen through the lens of closure theory, self-regulation was viewed as the monopolization of markets by means of protective legislation that provides the profession exclusive credentials and privileges with little regard for the public interest [22,31,32]). Freidson [33] argues that professional power stems from the professional monopoly over knowledge. In turn, this autonomy enables self-regulation and control over resources [34].

Admittedly, the firm opposition of many medical associations worldwide to the establishment of public health insurance plans over the twentieth century, including in Israel, largely confirms the approach that emphasizes self-interest as a chief motivation of organized medicine [35,36]. As Saks [23] aptly observes, the sociological critique of the medical profession coincides with the prevailing economic paradigm of the desirability of free market competition, thus viewing professional organizations as interest groups collectively dominating the market at the expense of public interest. Arguably, the increasing commercialization of medical care in the period after World War II made this institutionalized, altruistic, collective orientation more difficult to detect. This has led to a re-evaluation of the nature of the medical profession [37].

Hence, on the face of it, there is little reason to believe that medical associations will trouble themselves and invest valuable resources in engaging with public health issues but will instead pay them lip service on certain occasions when it is worth being portrayed as the public defender, doing so in a calculated manner, for their own self-interest. On the other hand, medical associations are comprised of individual members who, at least to some extent, consider themselves morally and professionally obligated to the public interest and expect their representative organization to act upon this obligation. While there is a lively discussion on trade unions’ nature as democratic entities that allow for the representation of all the desires and agendas of their members, scholars agree that the union’s own legitimacy is at least partially dependent on its ability to demonstrate democratic credentials. By the same token, effective trade unions depend on active participation from their members and their ability to mobilize and implement collective action [38,39]. Therefore, there is reason to assume that medical associations might consider social involvement as a strategic sphere of action to fulfill the wishes of their members. Presumably, however, the form in which their involvement takes shape will be derived not only from the altruistic desires of the members, but from the goal of these organizations to maximize the interests of the medical profession, especially when individual physicians lack the means to interfere in the political process and assess its outcomes, as will be further discussed.

In what follows, we will present our study’s design and method. Next, we will explore how physicians perceive their own role, as well as the role of their representative organizations, as agents of social change, and examine how this is reflected in the activities of these organizations.

## 2. Methods

This is a qualitative study based on in-depth interviews with senior official physicians in the Israeli healthcare system, and the mapping of the legislative, policy and public initiatives of Israeli physician associations during the span of a decade, between 2008 and 2018.

### 2.1. Interviews

Thirteen senior physicians were interviewed. They were selected using intentional sampling to represent a variety of medical specialties and healthcare sectors such as hospitals and community medicine. Prior to the start of the interview, the objectives of the study were explained to the interviewees. They were asked to sign a consent form agreeing to participate in the interview and to it being recorded. Table 1 describes the interviewees characteristics.

Semi-structured, in-depth interviews were conducted, allowing for flexibility and for questions to be raised in addition to those that were pre-formulated. Sometimes the wording and order of the questions were changed according to the dynamics of the interview. Questions included (but were not limited to): Do you think physicians should engage in public health activities outside the realms of the medical system? How can physicians be encouraged to take their place as leaders of social change in the field of public health? Do you think it is the role of medical associations to lead activities to promote public health? Can you tell us about public health initiatives in which you have been involved?

The interviews were recorded and transcribed verbatim. Anonymity was ensured. The authors analyzed the recorded and transcribed interviews, using a qualitative-phenomenological approach [40] in three stages. First, the interviews were read carefully, in order to understand the perceptions of the interviewees. Notes were written and key phrases in the text were marked in order to identify preliminary thematic categories in a process of data-driven, inductive, “bottom-up” thematic analysis. This means that the identified themes are strongly linked to the data themselves, without attempts to fit the data into a pre-existing theory or framework. To some extent, this method allows the researchers a certain degree of freedom from pre-existing concepts and presumptions regarding the subject in question [41,42].

In the second phase, the interviews were divided into categories, based on the identification of salient themes. In the third and final phase, these themes were organized into an integrative picture that described the interviewees’ perceptions of social leadership in the domain of public health. To capture the essence of this phenomena, the authors offered a description of the findings, accompanied by a detailed presentation of quotations from the interviewees. This provided evidence for a match between the interpretation (categorization of themes) and the distinctive voices of the interviewees, while balancing the meanings given by the interviewees (subjectivity) and the researchers’ interpretation (reflectivity), as suggested by Nutt-Williams and Morrow [43].

### 2.2. Mapping of Policy and Social Initiatives

The main sources of information used for the review are the websites of the Israeli Medical Association (IMA) and its medical societies (accessed 15 July 2020), media reports, and legal databases [44,45]. The IMA is the representative organization of Israeli physicians, operating as a professional, independent, and nonpartisan organization. The IMA is comprised of 52 scientific associations, each representing a medical branch that is a recognized specialty field in Israel, as well as various workgroups and societies dedicated to research and practice in specific medical domains.

The initiatives were categorized and examined according to the relevant field of policy (inequalities, nutrition, etc.), their purpose (restrictions on advertising, taxation, etc.) and, where possible, the extent of their success (for example, whether a bill was passed). Public engagement may be expressed, for example, in the promotion of bills or in orchestrating campaigns that engage with public health.

## 3. Results

### 3.1. Findings of the Interviews

Most interviewees held that physicians should engage in public activity, such as the promotion of healthcare policy, raising awareness of the needs of the healthcare system, legislative efforts, governmental relations, and advocacy. Many were aware of the aspects of volunteerism and the moral commitment associated with the concept of professionalism in medicine, as illustrated by the remarks of Interviewee no. 10:

“*I believe that being a physician is something greater than the medical practice. It is something that must have leadership for the sake of influence, quality of life, preventive medicine.” Interviewee no. 5 reinforced this view, noting that “physicians cannot claim innocence and immerse themselves only in hospitals and their work. Anyone who has that spark, the will to change things, must lead and take part in social, legislative, governmental processes.*”

Interviewee no. 2 shared his experience in voluntary activities: “*I regularly lecture in senior citizen clubs, health non-for-profit organizations and nursing homes on pharmaceuticals… Several physicians I know volunteer in the asylum-seekers clinic in Tel-Aviv*.”

On the other hand, there was evident tension between the personal desire of most physicians to focus on their medical practice and the need to engage more broadly in social issues as an inseparable aspect of the medical profession. Indeed, interviewees explained that, in practice, there is a gap between the real and the ideal in all aspects of social involvement by physicians. The healthcare system operates under constant conditions of scarcity and, therefore, compels all its participants, including physicians, to engage in a perpetual struggle to secure resources as well as their own position within the system. Under these circumstances, physicians lack the spare time and energy needed to be active in the public sphere. According to Interviewee no. 4, for example, “*I think that here, unfortunately, when there is a personnel shortage, people don’t feel that they have the energy to influence other areas. I think that this is quite a shame because the voice of physicians is very important*.”

Furthermore, interviewees explained that most physicians are not interested in public activism. They want to engage in the field in which they were trained. They also have an aversion to pursuing issues of a political nature, which at least some of the physicians perceived as “distasteful”. In addition, almost nothing in their medical training touched on issues that would enable physicians to engage seriously in matters of policy, legislation, or macro-economics. As such, even if they wish to become socially active, many physicians lack the necessary tools. As interviewee no. 1 said of his peers: “*They want to come to the ward in the morning, take care of their 30 patients as best they can, and that’s all. They do not want to argue. There is a lot of reluctance about these things.*”

According to this outlook, it is unrealistic to hope for mass mobilization toward social activism from physicians. Only a limited number of physicians are passionate about driving social initiatives beyond the walls of medical institutions. As interviewee no. 6 puts it: “*The term ‘fixation’ fits here very well*.” Essentially, they are private entrepreneurs of social change for whom the medical association can serve as a professional and organizational platform that allows them to take action and provides them with the support and tools to do so, such as channels of communication with Knesset members, the means of participating in Knesset committee discussions, and appearances in the media.

Interviewee no. 8 addressed the nature of modern medicine as a challenge to the broader understanding and involvement in social aspects of healthcare on the part of the physician, since physicians, at present, tend to specialize and invest themselves in very narrow medical fields: “*Unfortunately, nowadays we are less Renaissance people than before, because [medical] fields are sometimes so specific and delineated. It is difficult to master every subject, but yes, I have a kind of fantasy that physicians will receive a medical education that will go beyond that*.”

Some interviewees drew a distinction between the organizational level and that of the individual physician. In their view, the professional organization should operate on both “fronts”—representing the interests of its members as well as promoting health and the healthcare system. Interviewee no. 11: “*In my opinion, there is no doubt that a trade union, beyond strengthening the profession, is not only allowed, but it is its duty to express an opinion, and to try to influence in broader areas which are related to the profession.*”

Interviewee no. 2 reinforced this outlook: “*Just as the medical association is involved in regulating postgraduate medical education, it should also nurture social leadership [in regard to public health], with a focus on ethics and communication*.”

In practice, according to some interviewees, the two might clash, and, in fact, the organization is not always interested in or capable of addressing two fronts simultaneously, given its budgetary and personnel limitations. Nevertheless, it was noted during the interviews that the public campaign led by the Israel Society of Internal Medicine (ISIM) in 2019 to strengthen internal medicine departments within general hospitals is conceived as a successful initiative that gained wide public awareness. One interviewee cited a campaign against smoking as a major success. Conversely, another interviewee argued that the profession has had very limited success in social struggles, since, unlike security or education, the issue of healthcare has not become a national priority.

### 3.2. Engagement of Medical Associations with Socially Related Health Issues

The following items emerged as major issues in which the IMA and its medical societies invested resources and efforts to drive social change: health and healthcare disparities, healthy nutrition, the fight against smoking, and, to a lesser extent, rights of asylum seekers and those lacking resident status in Israel. The measures they employ are diverse and include legislative efforts, scientific activity, advocacy, lobbying, campaigning and so forth.

#### 3.2.1. Reduction of Health and Healthcare Inequalities

Scientific activity: In 2008, an IMA expert committee formulated a list of recommendations, such as a reduction in socio-economic inequality nationwide, investment in health care infrastructure in the periphery and minimization of economic barriers to health services [46]. In addition, the IMA has conducted a series of annual surveys focused on health disparities in Israel. These surveys include questions on issues such as the economic burden of health expenses on households and the propensity of individuals and families to forego medical treatment because of economic constraints [47].

Policy: Arguably, the most notable measure surrounding the issue of health disparities was the creation of financial incentives for medical residents who choose to train in peripheral hospitals, established under the 2011 physicians’ collective bargaining agreement [48]. There is controversy regarding the success of this measure. On the one hand, there are those who believe that the financial incentives influenced the movement of doctors to the periphery. On the other hand, there are those who believe that their impact was small [49,50]. Nevertheless, throughout the following decade, the preservation and expansion of these financial incentives were at the forefront of the agenda of the IMA and its scientific societies.

#### 3.2.2. Healthy Nutrition

The IMA has participated in the initiation and promotion of a legislative bill to amend the Public Health (Food) Ordinance by instituting the mandatory labelling of sugar and fat content. The proposed legislation is intended to provide the public with accessible information about the nutritional value of potentially harmful foods [51]. The proposed legislation was approved in 2017 and went into effect in 2020.

In 2014–2015, the IMA participated in a social involvement project in cooperation with Tel Aviv University, in which students who sought to promote civil society addressed a range of issues, including childhood obesity [52]. Under the IMA’s guidance, students researched the issue and examined ways of supporting the campaign against obesity through tools such as legislation and advocacy.

Another measure the IMA took in this area was to issue a declaration, in May 2018, identifying obesity as a disease. The initiative was led by the Israeli Association for the Study of Obesity (IASO), which operates under the auspices of the IMA. The purpose of this declaration was to recognize individuals who suffer from obesity as patients in need of medical assistance. Accordingly, the declaration has professional implications, such as the need to establish a national system for the treatment of obesity, including designated clinics in hospitals and the community. The declaration conflicts with the position adopted by the Israel Association of Public Health Physicians (IAPHP), which is also an IMA body, and which regards obesity as a physiological phenomenon that does not constitute a disease in itself but could be a contributing factor to other pathologies [53]. Moreover, according to this Association, the declaration reduces the phenomenon, including its social, cultural, and economic aspects, to a strictly medical issue and, in so doing, may actually undermine the campaign against obesity.

#### 3.2.3. Smoking Cessation

With the support of several Knesset members, and in cooperation with the IAPHP as well as the Israeli Society for the Prevention of Smoking (ISPS), the IMA initiated and promoted a legislative bill to prohibit smoking in children’s playgrounds [54]. The proposed legislation bans smoking near children in public playgrounds and within ten meters of nursery schools and kindergartens. In late 2017, this provision was adopted as an order of the Minister of Health, thereby establishing the prohibition [55].

The IMA has also initiated and promoted a legislative bill to amend the Prevention of Smoking in Public Places and Exposure to Smoking Law in relation to its enforcement at hospitals. The proposed legislation is intended to facilitate hospitals’ enforcement of the law and prevent their having to use their own budgets toward this end. The bill failed to pass a preliminary vote in the parliament.

Other measures include: An annual “Smoke-Free Day” at the Knesset, in cooperation with additional organizations and Knesset members. The day includes discussions and activities in furtherance of the campaign against smoking and in collaboration with Knesset members; The publication of a position paper calling on the government to strengthen the campaign against smoking [55]; A campaign for the taxation of tobacco products; Opposition to the introduction of new tobacco products in Israel; A formal letter to the Ministry of Health requesting that IQOS marketing and advertising be curtailed [56].

The ISPS and the IAPHP collaboratively conduct professional activities (lectures and conferences) aimed at encouraging the medical community to practice immediate intervention for the cessation of smoking among smokers.

#### 3.2.4. Healthcare for Asylum Seekers

Asylum seekers in Israel are not entitled to healthcare under the 1994 National Health Insurance Law and are only eligible for urgent medical treatment. The IMA has initiated proposed legislation to amend this law and extend its provisions to asylum seekers, with special attention to children [57]. The bill encountered the opposition of the government and was not further advanced [58].

In 2008, the IMA and the Ministry of Health participated in various forms of advocacy: The IMA appealed to the Ministry of Health to prevent the curtailment of medical care for asylum seekers and the status-less. A clinic was established for asylum seekers and the status-less in Tel Aviv. The clinic provides care through volunteer physicians [59]. Table 2 Summaries the medical associations’ activities in the public sphere.

## 4. Discussion

As both the set of interviews and the review of activities indicate, physicians and medical associations tend to engage in socially related health issues. Physicians feel a moral and professional commitment to do so as part of their profession. However, this does not mean that most physicians participate in such efforts. It appears that much of this activity is carried out by a few individuals, described in the interviews as having a “fixation”. The interviews indicate that there may be different reasons for this, including lack of will or interest, lack of suitable skills or knowledge, lack of time and resources, or an inclination toward conformity. These findings reveal a fairly wide gap between physicians’ stated positions and actions, and, to some extent, illustrate the inherent conflict between their professional ethos and neoliberal worldview [60]. Hence, it is possible to understand their expectation that their representative organization will fill the existing void and carry out public activities for the benefit of the public good. However, the transition from the individual level to the organizational level is accompanied by a transformation in the motivations of the agent of action.

The individual physician places the issue of public activity at the doorstep of her representative organization, which responds in turn. Indeed, it seems that the IMA deals with social issues extensively and continuously, while devoting considerable efforts to pushing them forward. The findings show that this is not just an isolated action of one kind or another in order to go through the motions. Consequently, public activity is largely dictated by the organization’s utilitarian considerations, as well as confined by them. This is mainly evident from the way the IMA tends to medicalize socially related health issues, that is, framing them as medical, not social, problems, and consequently offering an individual—not a political—remedy.

Scholars have long described medicalization as a process by which social problems are defined and treated as medical problems. Contemporary works have demonstrated how political actors and institutions promote the medicalization of such social problems, such as unintended pregnancies and unemployment [61,62,63]. In the Israeli case, this is perhaps best illustrated by the decision of the IMA to define obesity as a disease, despite the objection of the IAPHP, thus demonstrating the tension within the medical community itself between two very divergent worldviews. The first considers socio-political factors such as poverty and inequality to be the root of illness. Accordingly, the solution to containing morbidity also lies in the socio-political factors, and not necessarily at the individual medical level, which is focused on the recovery of the already-ill individual. In the words of Rudolph Wirchow, “Medicine is a social science, and politics is nothing more than medicine on a grand scale” [64]. This approach, based on “health in all policies” and “social medicine” approaches, is established within public health thinking, but still struggles to be adopted by most clinical physicians.

The second approach is that of the biomedical model, which focuses on medical solutions at the individual level rather than systemic change [65]. It is essentially based on providing a remedy to medical problems that largely stem from the existing political-economic order, and, therefore, it depends on this order and, in turn, reinforces it by “inventing” new problems or defining them as medical conditions that may be remedied at the individual level. Social medicine, in contrast, disrupts and threatens the existing order because it aspires to eradicate those problems, and thereby substantially reduce the economic activity they entail.

Social and environmental factors, such as education, income level, access to transportation and exposure to air pollution, account for approximately 50% of health outcomes and, moreover, play a large part in shaping human behavior—which is responsible for an additional 30% of health outcomes resulting from habits such as smoking, alcohol consumption, nutrition and exercise [66,67]. As such, one might expect organized medicine to invest most of its efforts in eradicating the sources of morbidity that are located “upstream” rather than expanding the healthcare services, which typically offer a “downstream” solution. However, healthcare is a significant industry and, in an Israeli context, a business enterprise with a private share amounting to almost 40% of national health expenditure [7]. The individual treatment of medical conditions is, therefore, a valuable source of income for practitioners, certainly a far more lucrative one than the eradication of these conditions in the first place. In total, obesity, overweight and sugar consumption accounted for approximately 2.5 billion NIS in direct treatment costs, that is, around 0.21% of the total Israeli gross domestic product (GDP). Coronary Artery Disease, type II diabetes and hypertension account for over three-fifths of the direct costs related to overweight alone [68]. Moreover, global survey data for 2014 show that Israel ranked first among OECD (Organisation for Economic Co-operation and Development) countries in performing bariatric surgery relative to population size, and second in the world. The profitability of these surgeries raises the concern that economic considerations might influence the decision to operate.

In line with this, the most noteworthy effort to reduce healthcare disparities across population groups in Israel in recent years was based on the provision of economic incentives for physicians to relocate from central Israel to peripheral areas, at a cost of approximately billion NIS over the course of roughly a decade—an effort that yielded questionable benefit [69]. This constituted another attempt to provide a medical solution to social problems, rather than improving the conditions of the population in rural and remote areas in the first place by reallocating national resources for education, culture, employment, transportation and so forth. From this perspective, it is no wonder that the initiative that was mentioned during the interviews as particularly successful by interviewees was the ISIM campaign, which was designed to promote internal medicine hospital departments.

However, the discourse of equality, led by the medical profession in its demand for economic incentives for physicians, leaves little room for a more thorough discussion on the desirable deployment of health services in Israel. Economies of scale may lead to the conclusion that the concentration of sophisticated medical services requiring extensive technological expertise, such as cardiac surgery and neurology, in several large tertiary care centers is preferable to their geographical dispersion, as opposed to primary care, which should be deployed in rural and remote areas.

In addition, when the IMA and its scientific societies engage in public health issues, they are decidedly inclined to focus on individual lifestyle changes, through a set of restrictions and prohibitions supplemented by punishment, such as fines for smoking, alongside efforts aimed at “public education” or “raising awareness”, such as nutritional labeling, which again place the responsibility on consumers regardless of their financial means or level of education.

Just as the biomedical model focuses on “rectifying” the individual rather than society, the efforts of political and social institutions to prevent morbidity focus on individual behavior rather than changing the existing order. In the spirit of the neoliberal approach that has been dominating Israel for decades [70], the responsibility for preventing disease is assigned to the individual through discourse and practices focused on “preventing behavioral risks” and “promoting a healthy lifestyle”, rather than advocating for the reduction in socio-economic disparities and increase in nutritional security, alongside challenging overconsumption or the notion of “economic growth” that, at least among wealthy countries, is becoming increasingly detached from indicators of health, quality of life and sustainability [71,72,73,74,75].

Therefore, the findings of this study suggest that medical associations tend to lead social changes, but they do so only if such processes do not challenge the prevailing neoliberal order or undermine the place of the medical profession within it. This is also evident in a comparison between the relatively large scope of activities in the areas of nutrition and smoking, in which great emphasis is placed on lifestyle change, and the paucity of activities in areas of social and environmental determinants of health that require profound systemic changes.

It appears that medical education requires a thorough transformation to integrate biosocial training with a view to ensuring that future physicians are equipped with the knowledge and skills needed to practice social medicine. Such tools could take the form of leadership programs, strengthening the relations between social organizations and healthcare providers and encouraging physicians to pursue an education in areas other than medicine. Such programs are already taking place across the globe, mainly in an intra-organizational leadership context [76,77]. In this context, the meaning of the term “asymmetric information” can be extended to describe knowledge gaps between practitioners and patients, in a social as well as clinical context.

Furthermore, it seems that, in this day and age, the involvement of health professionals in public affairs is of particular importance. Against the backdrop of the COVID-19 pandemic, while countries are experiencing an erosion of public confidence in state institutions [78], the involvement of the medical community in societal affairs, such as adhering to infection control measures and enhancing public trust in vaccination, is required perhaps more than ever.

## 5. Conclusions

This study aimed to explore what medical associations in Israel do to promote public health, what values underpin their activities, and how their actions can be interpreted. For this purpose, an analysis of both individual and organizational levels was applied, in an effort to yield a more nuanced understanding of the relationship between society and the medical profession. By so doing, we expected to elucidate not only the medical profession’s motivations and spheres of influence, but also its boundaries and limitations as an agent of social change.

The findings of this study revealed that, on the individual level, physicians believe in the importance of public activism, and expect their representative organizations to take action on this matter. However, on the organizational level, medical associations in Israel sometimes tend to construct social issues as medical challenges, while ignoring structural determinants in industrialized societies. These actions could be interpreted as an effort to shape social values and practices in a fashion that accommodates professional interests and consolidates medical dominance in wider society, and not necessarily to improve public health.

The impression that emerges from the interviews is that, from their perspective, physicians do indeed experience their activities as an effort to foster deep social change. Nevertheless, in practice, by depending on organized medicine to promote social change, they actually enhance the replication of neoliberal values. These coincide with the sectorial interests of the medical profession, striving to strengthen its social dominance and promote society’s dependency on medical services, or as Willis [79] put it—extend its sovereignty over the wider society. This point of departure eventually hampers physicians’ efforts to improve public health and makes it more difficult to create a meaningful social change.

Finally, this study demonstrates how institutional changes, that is, the retreat of state intervention on the one hand, and the entry of medical organizations into the public sphere on the other hand, may often promote processes of medicalization guided by market ideology. As a result, the field of health promotion and preventive medicine is moving away from the concept of social medicine and closer to the biomedical model.

## 6. Implications and limitations

Academic training in areas such as public policy, health systems management and public health could help physicians conceive of new modes of influence, while also developing a more critical perspective of the fundamental values of the current dominant neoliberal political and economic order. This can be achieved through the development of collaborative social leadership programs in cooperation with bodies such as the Ministry of Health, health funds, medical associations and various Civil–Society Organizations in the third sector.

This study has several limitations. The sample is relatively small (13 interviewees), as with the onset of the COVID-19 pandemic, physicians were no longer available to be interviewed. Furthermore, only senior physicians were interviewed. A wider range of views, especially from the next generation of trainee physicians, might have shed more light on the questions that this paper poses, especially when one of its main conclusions is the need for a change in medical education.

In addition, this study was limited to the investigation of the medical profession, although, in practice, engagement in public health is not exclusive to physicians, but common to the joint efforts of various health professionals. Future research may foster a more comprehensive perspective that accounts for the collaborative and holistic nature of public health.

Finally, one must take caution when drawing general conclusions from a single case study, especially when the unique organizational structure of the IMA is taken into account. As mentioned earlier, the IMA consists of scientific societies, unlike, for example, its counterparts in the United States, the United Kingdom, and other countries [11]. It can be assumed that, in an organizational framework such as this, it is not always possible to create a clear and absolute buffer between scientific activity and political processes when these are carried out under one roof. Differences in organizational settings, as well as in political and bureaucratic environments, may, therefore, lead to differences in medical associations’ attitudes and actions on issues such as public health and social engagement.

## Figures and Tables

**Table 1:** Characteristics of interviewees.

Characteristics	*N*
Gender	Nine men Four women
Role *	Two hospital directors One hospital deputy director Five heads of divisions at a hospital or in the Israel Ministry of Health One deputy director of Israel Ministry of Health division of health clinics (HMOs) Two heads of schools of medicine and public health One senior member of the Israel Medical Association (IMA) Four chairs of IMA union branches
Field of specialization *	5—Medical leadership 4—Public health 3—Internal health 2—Pediatrics 1—Ophthalmology (vision) 1—Surgery 1—Anesthesiology 1—Family medicine 1—Infectious disease
Description of Workplace *	6—Hospitals 4—Academia 3—Community health care 2—Ministry of Health
Sector	11—Jewish 2—Not Jewish

* Total is greater than the number of interviewees, as many have more than one position/specialization or place of employment. HMOs—Health Maintenance Organizations IMA—Israeli Medical Association.

**Table 2:** Summary of medical associations’ activities in the public sphere.

Subject	Goal	Main Actors	Channels	Tools	Main Results
Health and healthcare inequalities	Reduction in inequalities	IMA	Scientific activity, advocacy, policy formation	Publication of reports and surveys, financial incentives for physicians	Questionable benefit of financial incentives
Nutrition	Promotion of healthy nutrition	IMA; IASO; IAPHP	Legislation, education, public and scientific activity	Labeling harmful foods, learning activities on obesity, declaring obesity a disease	Legislation of food labeling passed; professional controversy over the declaration
Smoking	Smoking cessation	IMA; IAPHP; ISPS	Legislation, campaigns, advocacy, education	price elevation, bans, fines, lectures, workshops, limitation of import	Partial success in restricting smoking in public areas
Healthcare for asylum seekers	Granting medical rights and health insurance coverage	IMA	Legislation, advocacy, community involvement	Extension of healthcare coverage, provision of care on a voluntary basis	The bill was rejected, basic medical services given mainly on a voluntary basis

IMA—Israeli Medical Association. IASO—Israel Association for the Study of Obesity. IAPHP—Israeli Association of Public Health Physicians. ISPS—Israeli Society for the Prevention of Smoking.

## Data Availability

The data that support the findings of this study are available from the corresponding author.
